# Risk factors for emergency cesarean delivery in patients with placenta accreta spectrum: a multicenter retrospective cohort study

**DOI:** 10.3389/fmed.2025.1539998

**Published:** 2025-09-03

**Authors:** Min Li, Hongyuan Zhang, Xiao Song, Liang Li, Fanqing Meng, Ran Chu

**Affiliations:** ^1^Clinical Skills Training Center, Shandong Provincial Hospital Affiliated to Shandong First Medical University, Jinan, China; ^2^Department of Obstetrics and Gynecology, Shandong Provincial Hospital Affiliated to Shandong First Medical University, Jinan, China; ^3^Department of Obstetrics and Gynecology, Qilu Hospital of Shandong University Dezhou Hospital, Dezhou, China; ^4^Department of Anesthesiology, Qilu Hospital of Shandong University, Jinan, China; ^5^Department of Anesthesiology, Jinan Maternity and Child Care Hospital Affiliated to Shandong First Medical University, Jinan, China

**Keywords:** emergency cesarean, placenta accreta spectrum, placenta increta, placenta percreta, propensity score matching, perinatal outcomes, risk factor

## Abstract

**Background:**

Pregnant women with placenta accreta spectrum (PAS), particularly those with placenta increta or placenta percreta, undergoing emergency cesarean section are at a high risk of excessive intraoperative hemorrhage and related complications. This study aimed to evaluate maternal and neonatal outcomes and to identify risk factors associated with emergency cesarean section in women with PAS.

**Methods:**

A multicenter retrospective cohort study was conducted, including PAS patients who underwent cesarean section at three tertiary hospitals between January 2016 and January 2023. After 1:1 propensity score matching (PSM), clinical characteristics were compared between the emergency and elective cesarean section groups using chi-square tests and nonparametric rank-sum tests. Risk factors for emergency cesarean section were identified through Cox proportional hazards regression analysis.

**Results:**

Among 299 patients included in the study, 78 were selected for analysis after PSM. In the matched cohort, patients in the emergency cesarean section group required significantly more packed red blood cell transfusions (*p* = 0.034), had a higher rate of ascending branch ligation of the uterine artery (*p* < 0.001), required more neonatal intensive care unit admissions (*p* = 0.041), and delivered neonates with lower birth weight (*p* = 0.044). Key risk factors for emergency cesarean section included a history of more than one prior cesarean section [hazard ratio (HR), 2.34; 95% confidence intervals (CI): 1.24–4.42], preoperative hemoglobin levels ≤100 g/L (HR, 2.28; 95% CI: 1.19–4.40), preeclampsia (HR, 2.93; 95% CI, 1.10–7.82), and vascular lacunae within the placenta (HR, 0.40; 95% CI, 0.21–0.76).

**Conclusion:**

Emergency cesarean section in PAS patients is associated with increased transfusion requirements and adverse neonatal outcomes. Close monitoring and enhanced management of patients with identified risk factors may help improve maternal and neonatal outcomes.

## 1 Introduction

Placenta accreta spectrum (PAS) refers to a group of disorders characterized by abnormal adherence of the placental trophoblast to the uterine myometrium, encompassing three subtypes: placenta accreta, placenta increta, and placenta percreta (1). Over recent decades, the incidence of PAS has increased significantly, largely attributed to the rising rates of cesarean deliveries (2). As one of the most severe obstetric complications, PAS poses a significant threat to maternal health, primarily due to the failure of the placenta to detach from the uterus after delivery. This often results in life-threatening conditions such as disseminated intravascular coagulation (DIC), intensive care unit (ICU) admission, hysterectomy, and even maternal death (3).

Optimal management of PAS requires delivery in specialized centers equipped with multidisciplinary teams capable of providing comprehensive care. These centers must ensure immediate access to blood products, neonatal and adult intensive care facilities, and surgeons experienced in complex pelvic surgeries (4, 5). While clinical guidelines recommend planned delivery for PAS patients between 34 and 38 weeks of gestation, there remains no consensus on the optimal timing, as individual patient conditions vary significantly (6).

Our previous study revealed that approximately 10.8% of women with severe PAS and placenta previa required emergency cesarean delivery due to complications such as fetal distress, uterine contractions, or vaginal bleeding (7). Emergency cesarean delivery in these patients is strongly associated with increased intraoperative blood loss, higher maternal morbidity, and poor neonatal outcomes (8). Unlike planned cesarean deliveries, emergency procedures often lack sufficient time for preoperative preparation, including the assessment of maternal and fetal conditions, organization of blood transfusion protocols, and placement of prophylactic catheters or balloons in pelvic arteries to control hemorrhage. These limitations exacerbate surgical risks, resulting in higher rates of complications, such as massive blood transfusions, coagulation dysfunction, and urinary tract injuries (9).

Given these challenges, early and accurate risk assessment is crucial to identify PAS patients at high risk of requiring emergency cesarean delivery. This is particularly important in cases of placenta previa complicated by suspected placenta increta or percreta, where the likelihood of emergency interventions and adverse outcomes is significantly elevated.

In this multicenter retrospective cohort study, we utilized a propensity score matching (PSM) approach to compare perinatal outcomes between emergency and planned cesarean deliveries in PAS patients. Additionally, we sought to identify key risk factors associated with the need for emergency interventions. The findings of this study aim to support more individualized prenatal care strategies and improve maternal and neonatal outcomes in patients with PAS and placenta previa.

## 2 Materials and methods

### 2.1 General information

This retrospective cohort study included patients diagnosed with PAS (placenta increta or percreta) based on preoperative imaging, intraoperative findings, or postoperative pathological evaluation. All patients underwent pregnancy termination between January 2018 and June 2023 at four medical centers in Shandong Province, China: Shandong Provincial Hospital affiliated with Shandong First Medical University, Qilu Hospital of Shandong University, Jinan Maternity and Child Care Hospital affiliated with Shandong First Medical University, and Qilu Hospital of Shandong University Dezhou Hospital. All enrolled patients underwent uterus-preserving cesarean delivery, except those who required immediate hysterectomy due to uncontrollable intraoperative hemorrhage.

### 2.2 Patient variables

Patient clinical characteristics were extracted from medical records and included the following variables: maternal age at delivery, duration of labor, history of previous cesarean sections, obstetric complications, findings from prenatal obstetric ultrasonography, preoperative hemoglobin (HGB) levels, abdominal aorta balloon placement (BPAA), intraoperative hemostatic interventions, intraoperative blood loss, volume of transfused packed red blood cells (PRBC), perinatal outcomes, fetal birth weight, Apgar scores, and neonatal intensive care unit (NICU) admissions. Continuous variables were grouped according to either the median value in our study population or established clinical reference ranges to facilitate analysis and interpretation.

### 2.3 Definition and outcomes

The diagnosis of PAS in this study was primarily based on preoperative imaging assessments, including ultrasound and/or magnetic resonance imaging (MRI), and further confirmed by intraoperative findings observed by experienced obstetricians, with histopathological examination of the placenta conducted when available. The classification followed the criteria established by the International Federation of Gynecology and Obstetrics (FIGO) (1). According to FIGO, placenta percreta is characterized intraoperatively by grossly abnormal appearance of the placental bed, marked hypervascularity, and failure of placental separation during gentle cord traction, often accompanied by invagination of the uterine wall without visible serosal invasion; in contrast, placenta increta involves deeper myometrial invasion, with extension to the uterine serosa and potentially into adjacent pelvic structures, such as the bladder, broad ligament, vaginal wall, pelvic sidewall, or other pelvic organs (1). The diagnosis of placenta previa was confirmed by ultrasonographic examination performed after 16 weeks of gestation.

The primary outcome of this study was the occurrence of emergency cesarean delivery. For analytical purposes, the study population was categorized into two groups based on the timing and indication for delivery: the emergency cesarean delivery group and the planned cesarean delivery group. The emergency cesarean delivery group comprised patients who required urgent surgical intervention due to acute maternal or fetal complications. In contrast, the planned cesarean delivery group included patients who underwent cesarean section as scheduled, with no emergent indications. The most common indications for emergency cesarean delivery in this cohort included fetal distress, premature rupture of membranes, vaginal bleeding, uterine contractions, and uterine rupture.

### 2.4 Statistical analysis

Continuous variables were analyzed using the Mann–Whitney *U*-test, while categorical variables were compared using the chi-square test. A *p*-value of <0.05 was considered statistically significant.

PSM was applied to minimize selection bias between the emergency and elective cesarean delivery groups. Prior to performing PSM, preoperative characteristics that showed significant differences between the groups in the unmatched cohort (*p* < 0.05), as determined by chi-square tests, were identified and incorporated into a binary logistic regression model to calculate the propensity scores. Patients were matched 1:1 using the nearest-neighbor method with a caliper width of 0.2 standard deviations of the logit of the propensity score. The balance in propensity score distributions between groups, before and after matching, was assessed using scatter plots and histograms.

Cox proportional hazards regression analysis was conducted to evaluate associations between preoperative characteristics and the occurrence of emergency cesarean delivery, with labor duration as the timescale and emergency cesarean delivery as the censored event. Variables with a *p*-value <0.15 in univariate analysis were included in multivariate Cox regression models to identify independent risk factors. Results were presented as hazard ratios (HR) with 95% confidence intervals (CI). The cumulative risk of emergency cesarean delivery, stratified by identified risk factors, was estimated using Kaplan–Meier survival curves and compared using log-rank tests.

All statistical tests, including the Mann–Whitney *U*-test, chi-square test, and univariate and multivariate Cox proportional hazards regression analyses, were performed using IBM SPSS Statistics software (version 26.0). Propensity score matching and Kaplan–Meier survival analyses were performed using R software (version 4.1.2).

### 2.5 Ethics

All procedures carried out in studies involving human participants adhered to the ethical standards set by the institutional and national research committee, in accordance with the 1964 Helsinki Declaration and its subsequent amendments. The study received review and approval from the Ethical Committee of Qilu Hospital of Shandong University (protocol number KYLL-202309-028), with a waiver for informed consent.

## 3 Results

### 3.1 Preoperative characteristics before and after PSM

**Figure 1:**
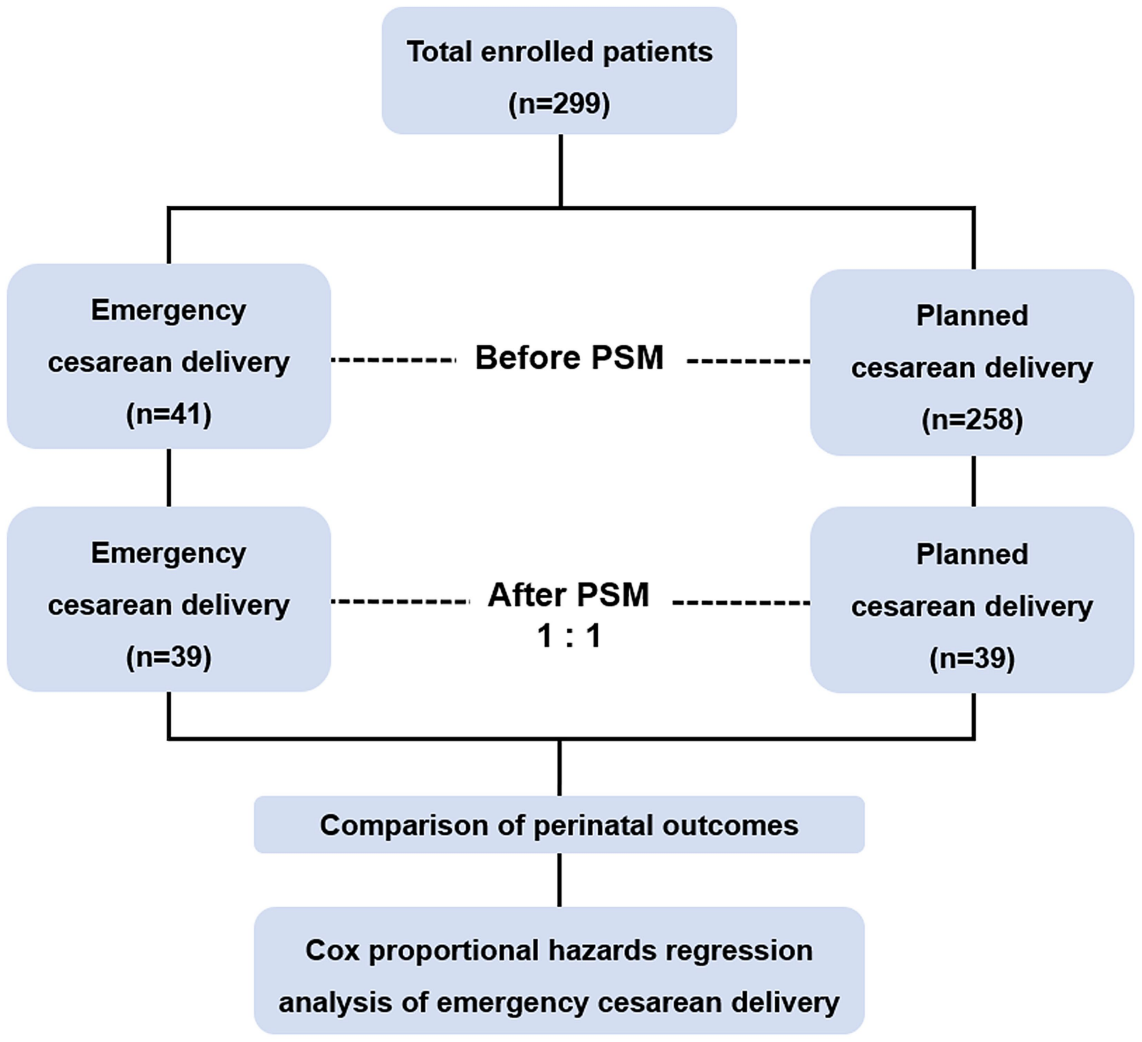
Flowchart of the study. PSM, propensity score matching.

Figure 1 provides an overview of the research procedure. A total of 299 women with placenta previa were included in this study, among whom 275 (92.0%) were diagnosed with placenta increta and 24 (8.0%) with placenta percreta. Table 1 summarizes the preoperative characteristics of the study population. Of these, 41 patients were categorized into the emergency cesarean delivery group, while 258 patients were assigned to the planned cesarean delivery group. Table 2 outlines the indications for emergency cesarean delivery. The most common indications were vaginal bleeding (*n* = 18, 43.9%) and uterine contractions (*n* = 11, 26.8%).

**Table 1:** Preoperative characteristics of patients before and after PSM.

Characteristics	Before matching				After matching			
	Total (*n* = 299)	Emergency (*n* = 41)	Planned (*n* = 258)	*P-*value	Total (*n* = 78)	Emergency (*n* = 39)	Planned (*n* = 39)	*P-*value
Age at delivery (years)				0.995				0.361
≤32	146 (48.8)	20 (48.8)	126 (48.8)		44 (56.4)	20 (51.3)	24 (61.5)	
>32	153 (51.2)	21 (51.2)	132 (51.2)		34 (43.6)	19 (48.7)	15 (38.5)	
History of uterine dilatation and curettage procedures				0.325				0.575
≤1	222 (74.2)	33 (80.5)	189 (73.3)		62 (79.5)	32 (82.1)	30 (76.9)	
>1	77 (25.8)	8 (19.5)	69 (26.7)		16 (20.5)	7 (17.9)	9 (23.1)	
Previous cesarean delivery				0.021				0.648
≤1	213 (71.2)	23 (56.1)	190 (73.6)		44 (56.4)	23 (59.0)	21 (53.8)	
>1	86 (28.8)	18 (43.9)	68 (26.4)		34 (43.6)	16 (41.0)	18 (46.2)	
Preoperative HGB level (g/L)				0.140				0.819
≤100	101 (33.8)	18 (43.9)	83 (32.2)		45 (57.7)	22 (56.4)	23 (59.0)	
>100	198 (66.2)	23 (56.1)	175 (67.8)		33 (42.3)	17 (43.6)	16 (41.0)	
Obstetric complications								
Preeclampsia	12 (4.0)	5 (12.2)	7 (2.7)	0.015	5 (6.4)	3 (7.7)	2 (5.1)	1.000
Gestational diabetes mellitus	38 (12.7)	3 (7.3)	35 (13.6)	0.264	6 (7.7)	3 (7.7)	3 (7.7)	1.000
Placenta previa classification				0.605				1.000
Marginal	41 (13.7)	7 (17.1)	34 (13.2)		14 (17.9)	7 (17.9)	7 (17.9)	
Partial	9 (3.0)	2 (4.9)	7 (2.7)		4 (5.1)	2 (5.1)	2 (5.1)	
Complete	249 (83.3)	32 (78.0)	217 (84.1)		60 (76.9)	30 (76.9)	30 (76.9)	
Prenatal ultrasound results								
Retroplacental myometrial thickness <1 mm	216 (72.2)	26 (63.4)	190 (73.6)	0.174	51 (65.4)	25 (64.1)	26 (66.7)	0.812
Vascular lacunae within the placenta	182 (60.9)	16 (39.0)	166 (64.3)	0.002	33 (42.3)	16 (41.0)	17 (43.6)	0.819
Hypervascularity of uterine-placental margin	206 (68.9)	22 (53.7)	184 (71.3)	0.023	44 (56.4)	22 (56.4)	22 (56.4)	1.000
Irregularity of uterine-bladder interface	82 (27.4)	8 (19.5)	74 (28.7)	0.221	12 (15.4)	8 (20.5)	4 (10.3)	0.209
Hypervascularity of the uterine serosa-bladder wall interface	96 (32.1)	11 (26.8)	85 (32.9)	0.436	18 (23.1)	11 (28.2)	7 (17.9)	0.282
Hypervascularity of cervix	43 (14.4)	5 (12.2)	38 (14.7)	0.668	8 (10.3)	5 (12.8)	3 (7.7)	0.711
Labor duration (weeks)				0.004				0.955
14 ≤ GW < 34	53 (17.7)	14 (34.1)	39 (15.1)		24 (30.8)	12 (30.8)	12 (30.8)	
34 ≤ GW < 37	138 (46.2)	19 (46.3)	119 (46.1)		39 (50.0)	19 (48.7)	20 (51.3)	
37 ≤ GW	108 (36.1)	8 (19.5)	100 (38.8)		15 (19.2)	8 (20.5)	7 (17.9)	

Values are *n* (%). HGB, hemoglobin; GW, gestational week.

**Table 2:** Indication for emergency cesarean section.

Indication	Emergency cesarean delivery (*n* = 41)
Vaginal bleeding	18 (43.9)
Uterine contractions	11 (26.8)
Uterine contractions with vaginal bleeding	5 (12.2)
Fetal distress	4 (9.8)
Premature rupture of membranes	2 (4.9)
Uterine rupture	1 (2.4)

Values are *n* (%).

In the unmatched cohort, women in the emergency cesarean delivery group were significantly more likely to have had a history of more than one prior cesarean delivery (43.9% vs. 26.4%, *p* = 0.021) and to have been diagnosed with preeclampsia (12.2% vs. 2.7%, *p* = 0.015) compared to the planned cesarean delivery group. Additionally, significant differences were observed in the presence of vascular lacunae within the placenta (*p* = 0.002), hypervascularity of the uterine-placental margin (*p* = 0.023), and labor duration (*p* = 0.004). To reduce potential bias between the two groups, propensity scores were calculated based on these preoperative characteristics.

**Figure 2:**
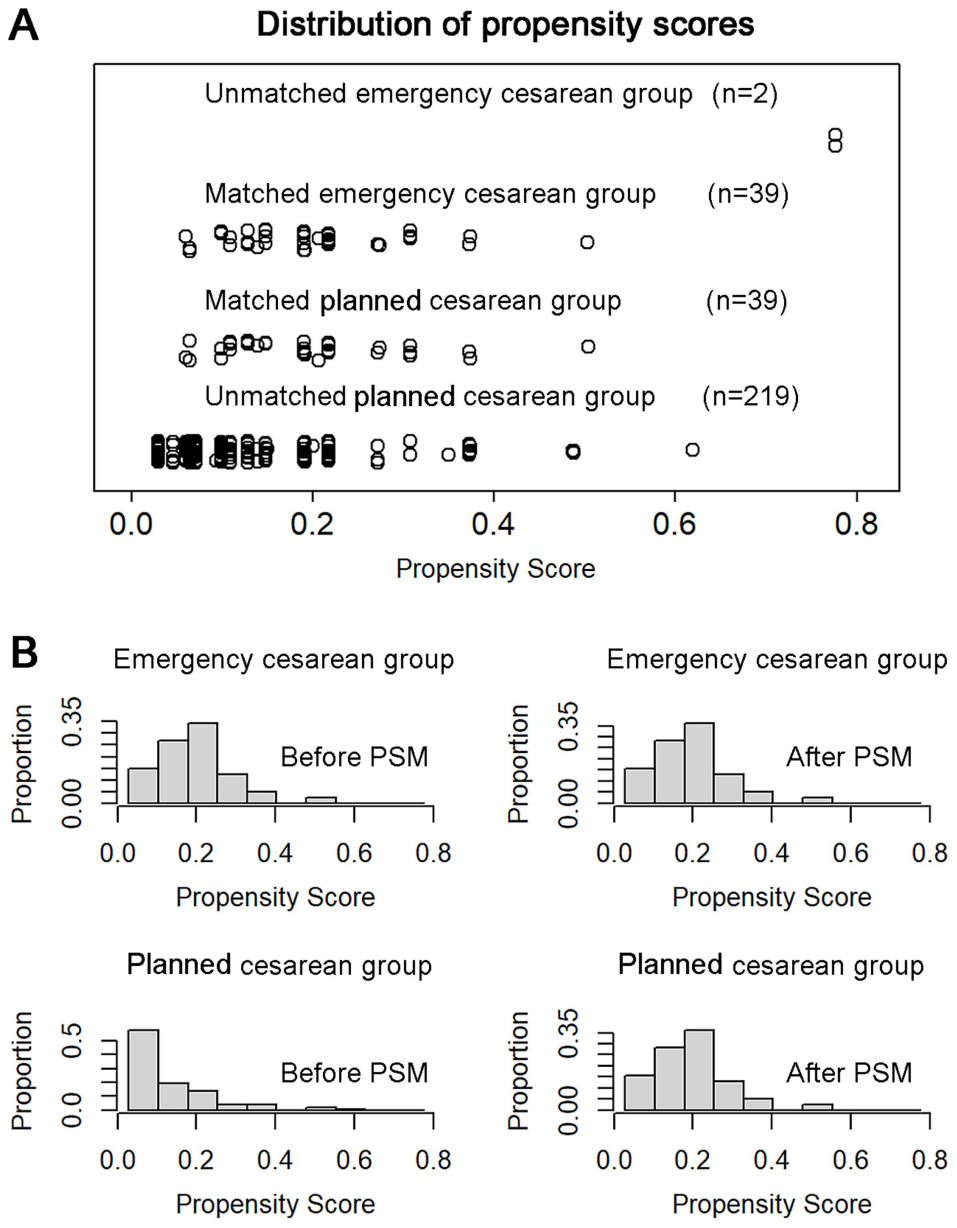
The distribution of propensity score before and after PSM analysis. **(A)** Thirty-nine patients in the emergency cesarean section group and 39 patients in the non-emergency group were successfully matched. **(B)** Histograms show the propensity scores of two groups are evenly more uniform distributed after matching. PSM, propensity score matching.

After PSM, 39 patients were included in each group. Post-matching analysis revealed no significant differences in preoperative characteristics between the emergency and planned cesarean delivery groups. Figure 2 illustrates the distribution of propensity scores before and after PSM, demonstrating that the scores were more evenly and uniformly distributed following matching.

### 3.2 Intergroup comparison of perinatal outcomes before and after PSM

A summary of perinatal outcomes is presented in Table 3. Before PSM, patients in the emergency cesarean delivery group received significantly more units of PRBC transfusions (median: 6 units vs. 4 units, *p* = 0.032), underwent B-Lynch suture more frequently (43.9% vs. 26.4%, *p* = 0.021), and were more likely to have ligation of the ascending branch of the uterine artery (29.3% vs. 14.0%, *p* = 0.013) or hysterectomy (9.8% vs. 1.6%, *p* = 0.014) compared to the planned cesarean delivery group. All hysterectomies were unplanned and performed as emergency procedures due to uncontrollable intraoperative hemorrhage. Additionally, the emergency group had lower fetal birth weights (median: 2,470 g vs. 2,900 g, *p* < 0.001) and a higher rate of neonatal intensive care unit (NICU) admissions (63.4% vs. 38.4%, *p* = 0.003).

**Table 3:** Comparisons of perinatal outcomes before and after PSM.

Perinatal outcomes			Before matching				After matching			
			Total (*n* = 299)	Emergency (*n* = 41)	Planned (*n* = 258)	*P-*value	Total (*n* = 78)	Emergency (*n* = 39)	Planned (*n* = 39)	*P-*value
Maternal										
Total operation time (min)			91 (74–120)	90 (77.5–147.5)	91.5 (73–117)	0.218	90 (75–125)	90 (75–150)	90 (72–120)	0.384
Length of hospital stay (days)			11 (8–17)	9 (6.5–16)	11 (8–18)	0.074	10 (7–18)	9 (7–17)	10 (8–18)	0.223
Postoperative length of hospital stay (days)			5 (4–7)	5 (4–8)	5 (4–7)	0.450	5 (4–7)	5 (4–8)	5 (4–7)	0.655
Intraoperative blood loss (mL)			1,500 (800–2,500)	1700 (800–3,300)	1,500 (800–2000)	0.123	1,500 (800–2,500)	1700 (800–3,400)	1,500 (600–2000)	0.086
Units of PRBC transfused			4 (4–8)	6 (4–12)	4 (3–8)	0.032	6 (2–8)	6 (4–12)	4 (2–8)	0.034
BPAA			74 (24.7)	4 (9.8)	70 (27.1)	0.017	10 (12.8)	4 (10.3)	6 (15.4)	0.498
B-Lynch suture			86 (28.8)	18 (43.9)	68 (26.4)	0.021	28 (35.9)	18 (46.2)	10 (25.6)	0.059
Ligation of ascending branch of uterine artery			48 (16.1)	12 (29.3)	36 (14.0)	0.013	12 (15.4)	12 (30.8)	0 (0.0)	<0.001
Tourniquet binding the lower uterine segment			50 (16.7)	6 (14.6)	44 (17.1)	0.700	15 (19.2)	6 (15.4)	9 (23.1)	0.389
Hysterectomy			8 (2.7)	4 (9.8)	4 (1.6)	0.014	3 (3.8)	3 (7.7)	0 (0.0)	0.240
Bladder repair			22 (7.4)	5 (12.2)	17 (6.6)	0.201	8 (10.3)	5 (12.8)	3 (7.7)	0.711
Systemic infections			7 (2.3)	0 (0.0)	7 (2.7)	0.599	2 (2.6)	0 (0.0)	2 (5.1)	0.494
Pulmonary embolism			1 (0.3)	0 (0.0)	1 (0.4)	1.000	0 (0.0)	0 (0.0)	0 (0.0)	-
DVT or thrombotic requiring therapy			2 (0.7)	0 (0.0)	2 (0.8)	1.000	0 (0.0)	0 (0.0)	0 (0.0)	-
DIC			3 (1.0)	1 (2.4)	2 (0.8)	0.359	0 (0.0)	0 (0.0)	0 (0.0)	-
ICU			4 (1.3)	2 (4.9)	2 (0.8)	0.092	2 (2.6)	2 (5.1)	0 (0.0)	0.494
Fetal										
Live birth			280 (93.6)	38 (92.7)	242 (93.8)	0.733	70 (89.7)	37 (94.9)	33 (84.6)	0.263
Apgar score (point)	1 min	0–7	52 (17.4)	11 (26.8)	41 (15.9)	0.086	21 (26.9)	10 (25.6)	11 (28.2)	0.799
		8–10	247 (82.6)	30 (73.2)	217 (84.1)		57 (73.1)	29 (74.4)	28 (71.8)	
	5 min	0–7	29 (9.7)	5 (12.2)	24 (9.3)	0.570	11 (14.1)	4 (10.3)	7 (17.9)	0.329
		8–10	270 (90.3)	36 (87.8)	234 (90.7)		67 (85.9)	35 (89.7)	32 (82.1)	
Weight (g)			2,850 (2450–3,200)	2,470 (1960–2,875)	2,900 (2500–3,200)	<0.001	2,645 (2275–3,100)	2,500 (2100–2,900)	2,900 (2400–3,200)	0.044
NICU			125 (41.8)	26 (63.4)	99 (38.4)	0.003	41 (52.6)	25 (64.1)	16 (41.0)	0.041
Death			20 (6.7)	4 (9.8)	16 (6.2)	0.496	8 (10.3)	3 (7.7)	5 (12.8)	0.711

Values are median (interquartile range) or *n* (%). PRBC, packed red blood cells; BPAA, balloon placement of abdominal aorta; DVT, deep vein thrombosis; DIC, disseminated intravascular coagulation; ICU, intensive care unit; NICU, neonatal intensive care unit.

After PSM, similar trends were observed. Patients in the emergency group required more units of PRBC transfusions (median: 6 units vs. 4 units, *p* = 0.034) and were significantly more likely to undergo ligation of the ascending branch of the uterine artery (30.8% vs. 0.0%, *p* < 0.001). Consistent with the findings before matching, the emergency group also had lower fetal birth weights (median: 2500 g vs. 2,900 g, *p* = 0.044) and a higher rate of NICU admissions (64.1% vs. 41.0%, *p* = 0.041) compared to the planned group.

### 3.3 High-risk factors associated with emergency cesarean delivery

**Figure 3:**
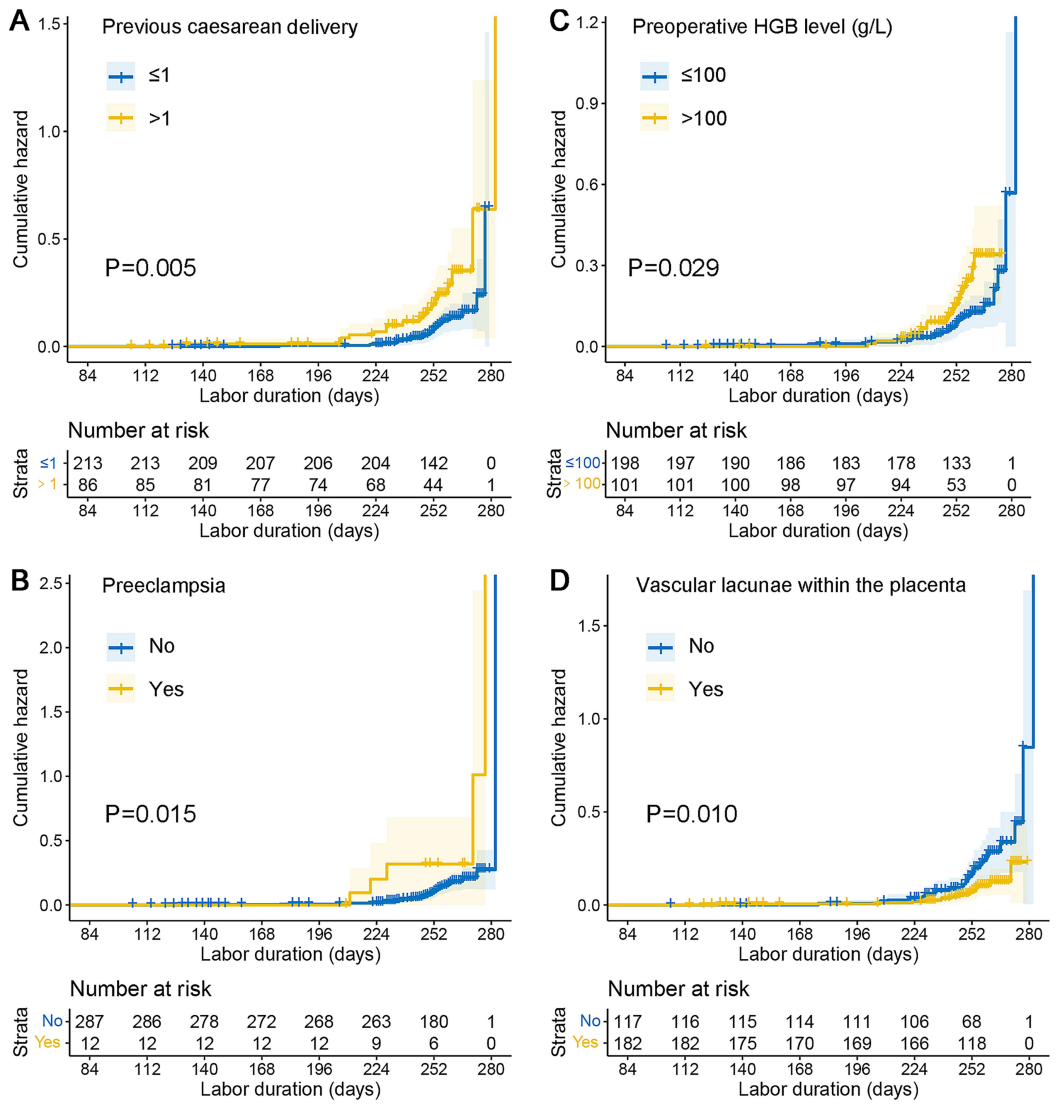
Kaplan–Meier cumulative risk curves emergency cesarean delivery. **(A)** Kaplan–Meier analysis curve of previous cesarean delivery. **(B)** Kaplan–Meier analysis curve of preeclampsia. **(C)** Kaplan–Meier analysis curve of preoperative HGB level. **(D)** Kaplan–Meier analysis curve of vascular lacunae within the placenta. HGB, hemoglobin.

The results of univariate and multivariate Cox proportional hazards regression analyses for risk factors associated with emergency cesarean delivery are summarized in Table 4. Multivariate analysis identified a history of more than one previous cesarean delivery (HR: 2.34, 95% CI: 1.24–4.42, *p* = 0.009), preoperative HGB level ≤100 g/L (HR: 2.28, 95% CI: 1.19–4.40, *p* = 0.013), and preeclampsia (HR: 2.93, 95% CI: 1.10–7.82, *p* = 0.032) as independent risk factors for emergency cesarean delivery. Conversely, the presence of vascular lacunae within the placenta was identified as a protective factor (HR: 0.40, 95% CI: 0.21–0.76, *p* = 0.005). Figure 3 illustrates the Kaplan–Meier cumulative risk curves, which demonstrate the cumulative likelihood of emergency cesarean delivery stratified by these high-risk factors.

**Table 4:** Univariable and multivariate Cox proportional hazards regression analysis for the premature emergency cesarean section before delivery.

Characteristics	Univariable		Multivariate	
	HR (95% CI)	*p*-value	HR (95% CI)	*p*-value
Age at delivery >32 years (vs. ≤32)	0.96 (0.52–1.78)	0.894		
History of dilatation and curettage of uterine >1 (vs. ≤1)	0.69 (0.32–1.49)	0.345		
Previous caesarean delivery >1 (vs. ≤1)	2.39 (1.27–4.47)	0.007	2.34 (1.24–4.42)	0.009
Preoperative HGB level ≤100 g/L (vs. >100)	2.00 (1.06–3.77)	0.033	2.28 (1.19–4.40)	0.013
Preeclampsia (vs. no)	3.11 (1.19–8.12)	0.021	2.93 (1.10–7.82)	0.032
Gestational diabetes mellitus (vs. no)	0.55 (0.17–1.78)	0.317		
Placenta previa classification		0.903		
Marginal	Reference	–		
Partial	1.45 (0.29–7.29)	0.651		
Complete	1.11 (0.46–2.70)	0.822		
Retroplacental myometrial thickness <1 mm (vs. no)	0.79 (0.41–1.53)	0.481		
Vascular lacunae within the placenta (vs. no)	0.44 (0.23–0.84)	0.012	0.40 (0.21–0.76)	0.005
Hypervascularity of uterine-placental margin (vs. no)	0.48 (0.26–0.89)	0.019		
Irregularity of uterine-bladder interface (vs. no)	0.68 (0.31–1.49)	0.338		
Hypervascularity of the uterine serosa-bladder wall interface (vs. no)	0.84 (0.42–1.69)	0.627		
Hypervascularity of cervix (vs. no)	0.86 (0.34–2.20)	0.754		

HR, hazard ratio; CI, confidence intervals; HGB, hemoglobin.

## 4 Discussion

This study highlights the significant perinatal risks associated with emergency cesarean delivery in patients with PAS and placenta previa. Emergency cesarean delivery was found to increase blood transfusion requirements, the need for additional surgical interventions, lower neonatal birth weights, and higher rates of NICU admissions. Multivariate Cox proportional hazards analysis identified multiple previous cesarean deliveries, preoperative hemoglobin levels ≤100 g/L, preeclampsia, and placental vascular lacunae as independent risk factors for emergency cesarean delivery. These findings emphasize the importance of early diagnosis and risk stratification. Enhanced prenatal surveillance, especially in patients identified as high-risk via ultrasound or MRI, is essential. Optimizing delivery planning at specialized medical centers with multidisciplinary teams experienced in PAS management can reduce the likelihood of emergency interventions and improve maternal and neonatal outcomes.

Early prenatal diagnosis of PAS remains critical for facilitating planned deliveries at specialized medical centers with experienced multidisciplinary teams, which can significantly reduce maternal morbidity (10). The primary goal in PAS management is to achieve a planned delivery, thereby minimizing the likelihood of emergency cesarean delivery and its associated complications (11). Our obstetrics department functions as a specialized medical center with a highly experienced multidisciplinary team in the diagnosis and treatment of PAS. Despite this, 13.7% (*n* = 41) of PAS patients in our study required emergency cesarean delivery due to complications such as vaginal bleeding, uterine contractions, fetal distress, premature rupture of membranes, or uterine rupture. Compared to planned cesarean deliveries, emergency procedures were associated with increased risks of severe hemorrhage, anesthetic complications, and inadvertent injuries to abdominopelvic organs (12). According to the American College of Obstetricians and Gynecologists, the standard time from the decision to perform an emergency cesarean delivery to the delivery of the infant should not exceed 30 min (13). This narrow time frame necessitates the implementation of specialized emergency measures, such as advanced preparation for massive transfusions, to reduce adverse maternal and neonatal outcomes and patient mortality. Early identification of PAS patients at high risk for emergency cesarean delivery is therefore crucial for improving clinical management and reducing the occurrence of adverse events.

Intraoperative and postoperative hemostatic strategies play a critical role in managing severe hemorrhage associated with PAS, especially during emergency cesarean delivery. In addition to standard surgical and interventional techniques, the use of pharmacological agents such as recombinant activated factor VII (rFVIIa) has been reported as an effective adjunct in controlling intractable postpartum bleeding. Recent evidence suggests that rFVIIa can significantly reduce blood loss and improve maternal outcomes in cases of massive obstetric hemorrhage, without substantially increasing thromboembolic risk (14). Incorporating such targeted hemostatic therapies into comprehensive surgical protocols may enhance bleeding control and reduce the need for hysterectomy or other radical procedures.

In our study, a comparison of the pre- and post-matching cohorts revealed that emergency cesarean delivery resulted in significantly higher PRBC transfusion requirements, consistent with findings from previous studies (15). Patients in the emergency group also required additional surgical interventions, such as ligation of the ascending branch of the uterine artery. Similarly, a retrospective study by Pires-Menard et al. (16) found that emergency cesarean delivery was associated with worse neonatal outcomes, with low birth weight being an independent risk factor for poor neonatal condition at birth. In our analysis, neonates in the emergency group had lower birth weights and higher NICU admission rates, even though the gestational age at delivery did not differ significantly between the groups. These findings reaffirm the adverse impact of emergency delivery on neonatal outcomes in PAS patients.

Our analysis of high-risk factors for emergency cesarean delivery differs from previous studies in two significant aspects. First, we deliberately excluded antepartum vaginal bleeding from the risk factor analysis, as it is a strong predictor of preterm delivery and adverse pregnancy outcomes in PAS patients (17, 18). This exclusion was intended to prevent the overshadowing of other potential risk factors, a methodological departure from prior studies (19). Second, instead of using logistic regression to analyze binary outcomes, we employed Cox proportional hazards regression analysis. This approach allowed us to evaluate the timing and severity of emergency deliveries, providing a more nuanced understanding of their association with various factors.

Consistent with previous research, our study confirmed that a history of more than one previous cesarean delivery significantly increases the risk of emergency cesarean delivery in PAS patients (19, 20). Preeclampsia also emerged as a significant risk factor, reflecting its well-documented association with adverse maternal and neonatal outcomes (21). Furthermore, our findings indicate that preoperative HGB levels ≤100 g/L are associated with heightened risks of emergency delivery and adverse outcomes. This observation aligns with our previous research, which linked low HGB levels to an increased likelihood of excessive bleeding during cesarean delivery. Additionally, vascular lacunae within the placenta were identified as a protective factor, potentially signaling early recognition of severe PAS and prompting timely delivery planning (8). In our analysis, the presence of placental vascular lacunae on prenatal ultrasound was associated with a lower risk of emergency cesarean section in patients with PAS. Although this result initially seemed counterintuitive—since vascular lacunae are often linked to more severe disease—it is likely related to differences in clinical management. In practice, identifying vascular lacunae tends to prompt closer monitoring and earlier planned cesarean delivery, which may reduce the likelihood of emergency interventions. Conversely, patients without vascular lacunae might be managed more conservatively, making them more susceptible to unexpected complications requiring emergency surgery. This finding underscores how clinical decision-making can influence outcomes and highlights the importance of management strategies in interpreting retrospective data. However, we acknowledge the possibility of residual confounding and detection bias. Further studies with larger sample sizes and more detailed clinical data are needed to clarify this association.

A major strength of our study lies in the use of Cox proportional hazards regression analysis to identify high-risk factors for emergency cesarean delivery in PAS patients. However, the study’s retrospective design inherently limits the availability of certain prenatal medical data. Moreover, our definition of emergency cesarean delivery encompassed various clinical scenarios, including fetal distress, premature rupture of membranes, vaginal bleeding, uterine contractions, and uterine rupture. The incidence of emergency cesarean delivery in our study (13.7%) was lower than that reported in previous studies, which may reflect differences in study populations or healthcare settings.

## 5 Conclusion

Emergency cesarean delivery in PAS patients significantly increases maternal and neonatal risks, including higher transfusion requirements, additional surgical interventions, and adverse neonatal outcomes. Independent risk factors such as multiple previous cesarean deliveries, preoperative hemoglobin ≤100 g/L, preeclampsia, and placental vascular lacunae highlight the need for early risk stratification and planned delivery at specialized centers to optimize outcomes in this high-risk population.

## Data Availability

The raw data supporting the conclusions of this article will be made available by the authors, without undue reservation.
